# Supplementary material for: Positive appraisal style predicts long-term stress resilience and mediates the effect of a pro-resilience intervention
Source: Nat Commun. 2025 Nov 21;16:10269. doi: 10.1038/s41467-025-65147-7 (PMC12638893; doi:10.1038/s41467-025-65147-7)
Supplement: Supplementary file 1 — Supplementary Files [file 41467_2025_65147_MOESM1_ESM.pdf]

Positive appraisal style predicts long-term stress resilience and mediates the effect of a pro-resilience intervention

SUPPLEMENTARY FILES

Papoula Petri-Romão[1], Roberto Mediavilla[2,3,4,*], Alexandra Restrepo-Henao[5,6], Lara MC Puhlmann[1,7], Matthias Zerban[1,8], Kira F Ahrens[9,10], Corrado Barbui[11], Ulrike Basten[12], Carmen Bayón[2,3,13,14], Andrea Chmitorz[15], Mireia Felez-Nobrega[16,17], Bianca Kollmann[1,29], Klaus Lieb[1,18], David McDaid[21], Kerry R McGreevy[2,3], Maria Melchior[20], Ainoa Muñoz-Sanjosé[2,3,13,14], Rebecca J Neumann[9], A-La Park[19], Michael M. Plichta[9], Marianna Purgato[11], Andreas Reif[9], Charlotte Schenk[9], Anita Schick[23], Alexandra Sebastian[1,18], Marit Sijbrandij[24], Pierre Smith[25,26], Oliver Tüscher[1,18-20], Michèle Wessa[1,27-29], Anke B Witteveen[24], Kenneth SL Yuen[1,8], Josep Maria Haro[3,17,20,30], José Luis Ayuso-Mateos[2,3,4,31,+], and Raffael Kalisch[1,8,+]

1. Leibniz Institute for Resilience Research, Mainz, Germany
2. Department of Psychiatry, Universidad Autónoma de Madrid (UAM), Madrid, Spain
3. Centro de Investigación Biomédica en Red en Salud Mental (CIBERSAM), Instituto de Salud Carlos III, Madrid, Spain
4. Instituto de Investigación Sanitaria del Hospital Universitario La Princesa (IIS-Princesa), Madrid, Spain
5. Department of Epidemiology, Columbia University Mailman School of Public Health, New York, NY, USA
6. Epidemiology Group, National School of Public Health, University of Antioquia, Medellín, Colombia
7. Clinical Psychology and Behavioural Neuroscience, Faculty of Psychology, Technische Universität Dresden, Dresden, Germany
8. Neuroimaging Center (NIC), Focus Program Translational Neuroscience (FTN), Johannes Gutenberg University Medical Center, Mainz, Germany
9. Department of Psychiatry, Psychosomatic Medicine and Psychotherapy, University Hospital Frankfurt, Frankfurt, Germany
10. Goethe University Frankfurt, Cooperative Brain Imaging Center - CoBIC, Frankfurt, Germany
11. WHO Collaborating Centre for Research and Training in Mental Health and Service Evaluation, Department of Neurosciences, Biomedicine and Movement Sciences, Section of Psychiatry, University of Verona, Verona, Italy
12. Department of Psychology, RPTU University of Kaiserslautern-Landau, Landau, Germany
13. Department of Psychiatry, Clinical Psychology, and Mental Health, Hospital Universitario La Paz, Madrid, Spain
14. Instituto de Investigación Sanitaria del Hospital Universitario La Paz (IdiPAZ), Madrid, Spain
15. Faculty of Social Work, Health Care and Nursing Science, Esslingen University of Applied Sciences, Esslingen, Germany
16. Group of Epidemiology of Mental Disorders and Ageing, Sant Joan de Déu Research Institute, Esplugues de Llobregat, Barcelona, Spain
17. Research, Teaching, and Innovation Unit, Parc Sanitari Sant Joan de Déu, Sant Boi de Llobregat, Barcelona, Spain
18. Department of Psychiatry and Psychotherapy, Johannes Gutenberg University Medical Center, Mainz, Germany
19. Department of Psychiatry, Psychotherapy and Psychosomatic Medicine University Medicine Halle (Saale) of the Martin Luther University Halle-Wittenberg (MLU)

20. German Center for Mental Health (DZPG), partner site Halle-Jena-Magdeburg
21. Care Policy and Evaluation Centre, Department of Health Policy, London School of Economics and Political Science, London, UK
22. Sorbonne Université, INSERM, Institut Pierre Louis d'Epidémiologie Et de Santé Publique (IPLESP), Équipe de Recherche en Épidémiologie Sociale (ERES), Paris, France
23. Department of Public Mental Health, Central Institute of Mental Health, Medical Faculty Mannheim, Heidelberg University, Mannheim, Germany
24. Department of Clinical, Neuro- and Developmental Psychology, WHO Collaborating Center for Research and Dissemination of Psychological Interventions, Amsterdam Public Health Research Institute, Vrije Universiteit Amsterdam, Amsterdam, The Netherlands
25. Department of Epidemiology and public health, Sciensano, Brussels, Belgium
26. Institute of Health and Society (IRSS), Université catholique de Louvain, Brussels, Belgium
27. DKFZ Hector Cancer Institute at the University Medical Center Mannheim, Germany
28. German Cancer Research Center (DKFZ) Heidelberg, Division of Cancer Survivorship and Psychological Resilience, Germany
29. Central Institute of Mental Health, Department of Neuropsychology and Psychological Resilience Research, Mannheim, Germany
30. Department of Medicine, Universitat de Barcelona, Barcelona, Spain
31. Department of Psychiatry, Hospital Universitario La Princesa, Madrid, Spain

+Equal contribution

*Corresponding author:

Roberto Mediavilla
Department of Psychiatry, Universidad Autónoma de Madrid
Arzobispo Morcillo, 4, 28029, Madrid (Spain)
roberto.mediavilla@uam.es
Tel:+34 91 497 24 47

Table of contents

Supplementary Notes ... 5

Supplementary Note 1. Theoretical supplementary material (Introduction) ... 5

Supplementary Note 2. Theoretical supplementary material (Discussion) ... 5

Supplementary Note 3. Supplementary Methods (Observational discovery sample MARP) ... 6

Observational discovery sample: MARP ... 7

Supplementary Table 1. MARP: demographics. ... 7

Supplementary Table 2a. MARP: Life events (LE) exposure in the analyzed sample from B0 to B2. . 9

Supplementary Table 2b. MARP: Daily hassles (DH) exposure in the analyzed sample from B0 to B2. ... 9

Supplementary Table 3. MARP: Prediction of stressor reactivity (SR) by PASS-content and PASS-process in the most stressor-exposed participants (top two terciles of mean E between B0 and B2), controlling for baseline (B0) covariates. ... 12

Supplementary Table 4. MARP: Prediction of stressor reactivity (SR) by perceived social support and perceived good stress recovery, controlling for baseline (B0) covariates. ... 13

Supplementary Table 5. MARP: Prediction of stressor reactivity (SR) by perceived social support and perceived good stress recovery in the most stressor-exposed participants (top two terciles of mean E between B0 and B2), controlling for baseline (B0) covariates. ... 14

Supplementary Table 16. MARP: Results of covariate selection. ... 15

Observational replication sample: LORA ... 16

Supplementary Table 6. LORA: demographics. ... 16

Supplementary Table 7a. LORA: Life events (LE) exposure in the analyzed sample from B0 to B2. 18

Supplementary Table 7b. LORA: Daily hassles (DH) exposure in the analyzed sample from B0 to B2. ... 18

Supplementary Table 8. LORA: Prediction of stressor reactivity (SR) by perceived social support and perceived good stress recovery, controlling for baseline B0) covariates. ... 21

Supplementary Table 9. LORA: Prediction of stressor reactivity (SR) by PASS-content and PASS-process in the most stressor-exposed participants (top two terciles of mean E between B0 and B2), controlling for baseline (B0) covariates. ... 22

Supplementary Table 10. LORA: Prediction of stressor reactivity (SR) by perceived social support and perceived good stress recovery in the most stressor-exposed participants (top two terciles of mean E between B0 and B2), controlling for baseline (B0) covariates. ... 23

Supplementary Table 17. LORA: Results of covariate selection ... 24

Interventional sample: RESPOND-RCT Spain ... 25

Supplementary Figure 1. CONSORT Flow Diagram of RESPOND-RCT Spain. ... 25

Supplementary Table 11. RESPOND-RCT Spain: Life events (LE) list. ... 26

Supplementary Table 12. RESPOND-RCT Spain: Daily hassles (DH) lists. ... 27

Supplementary Table 13. RESPOND-RCT Spain: demographics and group comparison. ... 28

Supplementary Table 14. RESPOND-RCT Spain: Stressor exposure per assessment time point and category (means and standard deviations). 30

Supplementary Table 15a. RESPOND-RCT Spain: Effect of the intervention on SR. 31

Supplementary Table 15b. RESPOND-RCT Spain: Effect sizes for the effect of the intervention on E, P and PAS, as well as the effect of PAS at baseline on SR. 31

Supplementary Table 18. RESPOND-RCT Spain: Results of covariate selection. 33

Supplementary Figure 2. RESPOND-RCT Spain: Directed acyclic graph showing the hypothetical causal pathways. 34

Supplementary Table 19. RESPOND-RCT Spain: Comparison of mediation models. 35

References 36

Supplementary Notes

Supplementary Note 1. Theoretical supplementary material (Introduction)

A central and integral claim of positive appraisal theory of resilience (PASTOR) is that PAS mediates the effects of other resilience factors on resilience [1]. This claim is based on the idea that any resilience factor will ultimately benefit resilience because it shapes the way one typically appraises stressors (potential threats to one's goals or needs) in a more positive way, and this is eventually what helps people stay mentally healthy despite adversity. In Kalisch et al.[1], section 4.2.4.2, we have given the explicit example of social support as a more "distal" resilience factor and have posited that a person who perceives themself as being well supported will usually perceive stressors as less threatening, for instance because they know that their support network would provide them with coping resources if needed. We also there argue that social support may have the additional effect of reducing one's actual stressor exposure, because helpers may take some burden off one's shoulders (see Figure 4A in Kalisch et al.[1]). Critically, when resilience is operationalized via the stressor reactivity (SR) score on the basis of extensive stressor and mental health monitoring, this latter effect is covered by the inclusion of the stressor exposure variable into the outcome (SR), which corrects for individual differences in exposure. Provided such correction, the only remaining statistically visible effect of social support on resilience should be via appraisal, according to the theory (Figure 4B in Kalisch et al., 2015). The example illustrates why and how PAS is posited to be a "proximal" resilience factor, that is, an immediate causal factor in the effect path towards resilience and why and how it is thought to integrate the effects of other resilience factors.

Analogous arguments apply to other resilience factors than social support (Kalisch and colleagues[1], 4.2.4.2 uses the examples of life history and genotype). To illustrate this in an exemplary fashion for other potential resilience factors that are presumably positively affected by the intervention in the RESPOND-RCT Spain: If one has better problem-solving techniques at one's disposal as a result of the intervention, this will a) shape one's appraisal of one's coping potential positively (enhancing PAS), and it will b) reduce stressor exposure because problems get solved more efficiently (the effect on exposure being factored out via the SR score). If one learns to act more in agreement with one's values and is kinder, this will a) reduce the perception of one's own actions as threats to one's self-esteem (one of the strongest stressors for most people) and hence enhance PAS, and it may b) also increase or decrease actual stressor exposure (e.g., increase because one avoids social conflicts less), but this will in any case be factored out through the SR score.

In sum, PASTOR predicts an effect of any intervention on resilience via PAS, no matter what the exact ingredients of the intervention are.

Supplementary Note 2. Theoretical supplementary material (Discussion)

PASTOR posits that in the case of milder stressors, initial (primary, fast, often unconscious and non-verbal) appraisals may sometimes be positive, such that the overall appraisal outcome is also positive[1]. This is termed "positive situation classification" and is believed to partly originate from positive experiences with similar stressors in the past or the application of cultural stereotypes to such mild stressor situations. In the case of more severe stressors, most initial appraisals are however believed to be negative, reflecting a natural default setting of our aversive system, which favors a cautious approach to possible threats ("better safe than sorry"). In these cases, to produce positive appraisal outcomes, secondary (slower, often conscious and verbal) re-appraisals are necessary, which may include an assessment of coping potential. Note that most of the items in the PASS-process questionnaire can be interpreted as reflecting such secondary appraisals.

Supplementary Note 3. Supplementary Methods (Observational discovery sample MARP)

Data cleaning included an investigation of the number and dates of online monitorings (T1, T2, …) and the dates of the battery administrations (B0, B1, B2). Outliers were defined as those participants that had less than 18 or more than 24 months between subsequent battery administrations (B0 to B1 or B1 to B2) and less than 36 or more than 48 months between B0 and B2. For these participants, the dates of the online monitorings and battery administrations, entered by the participants at the time of questionnaire completion, were double-checked against digital and paper records. This was done for 75 participants and altogether 216 assessments. 32 assessments (14.8%) needed correction based on the actual digital time stamp or date of a signature. Mistakes were likely due to typos in manually entered dates by participants. This error rate was deemed acceptable and all other outliers in length of time between assessment were considered genuine since the design of the study allowed it.

Observational discovery sample: MARP

Supplementary Table 1. MARP: demographics.

Variables		Mean	St. Dev.	Min	Max	n	n in %
age in years		19.15	0.815	17	21		
sex	female					83	62.9%
	male					49	37.1%
relationship status	single					131	99.2%
	registered civil same-sex partnership, living separately					1	0.8%
employment status	full-time employed					5	3.8%
	part-time employed					3	2.3%
	low-income employment, 400-euro job, mini-job					44	33.3%
	occasionally or irregularly employed					19	14.4%
	in vocational training					7	5.3%
	Military service/civilian service					1	0.8%
	voluntary social year					2	1.5%
	maternity leave, parental leave or other leave of absence					1	0.8%
	not employed (including: pupils or students not working for money, unemployed, early retirees, pensioners without additional income)					50	37.9%
education	student at a full-time general education school					8	6.1%
	Secondary school leaving certificate (high)					7	5.3%
	A-levels, graduation from a specialised secondary school					2	1.5%

	A-levels					113	86.3%
	A-levels via second educational pathway					1	0.8%
student status	student					92	69.7%
	no student					40	30.3%
mental illnesses in the family	yes					52	39.4%
	no					80	60.6%
monthly income		562.66	393.62	0	1700		
monthly household income		3562.78	7206.96	0	70000		
smoking[1]		0.41	1.95	0.00	15.00		
weed[2]		0.34	1.07	0	8		
life events score[3]		9.10	4.46	0	22		
childhood trauma[4]		1.31	0.34	1.00	2.96		
number of assessments[5]		12.81	1.23	10	18		

Note: [1] Number of cigarettes per day/month; [2] Times per month; [3] Summary of life events score; [4] Childhood trauma questionnaire (CTQ); [5] Number of assessments between the respective baselines St. Dev = standard deviation, Min = minimum, Max = maximum.

Supplementary Table 2a. MARP: Life events (LE) exposure in the analyzed sample from B0 to B2.

Variables	Average report (Mean)	Average severity (Mean)
Lost job	0.06	2.81
Traumatic incident at work	0.06	3.58
Wedding planning	0.03	2.17
Bought or sold a house, moving house	0.19	3.2
Major house renovation	0.07	2.78
Had something stolen or vanished	0.02	1.39
Legal problems	0.1	2.11
Serious financial problems	0.09	3.44
Serious illness, accident or diagnosis of oneself or a close family member (child, parent, sibling, grandparent)	0.23	3.71
Serious illness, accident or diagnosis of a close friend or partner	0.07	3.53
Death of a family member	0.08	3.8
Death of a friend (other than boyfriend/girlfriend)	0.03	3.89
Death of a beloved pet	0.03	3.93
Parents separated	0.02	3.65
Constant arguments between family members	0.31	3.42
Broke up with boyfriend/girlfriend or spouse	0.09	3.95
Serious arguments with boyfriend/girlfriend or spouse	0.2	3.96
Serious problems in relationships with friends	0.21	3.6
Child started school	0	2
Increased care for elderly or ill person	0.07	2.93
You/a partner had an abortion	0	NaN
Serious physical illness – unable to work or carry out normal activities	0.06	3.86
Injury – unable to work or carry out normal activities	0.07	3.7
You/a partner had a difficult pregnancy or miscarriage	0	NaN
Been physically assaulted or mugged	0	NaN
Been sexually assaulted	0	NaN
Other impactful event (e.g. exam, car accident, house fire, earthquake, war)	0.47	3.76

Supplementary Table 2b. MARP: Daily hassles (DH) exposure in the analyzed sample from B0 to B2.

Variables	Average days	Average severity
Losing or misplacing items	2.33	2.45
Negative event in the media	3.28	2.75
Negative political event	2.66	2.7
Social obligation	3.16	2.51
Interruption in activity (e.g., work or leisure)	3.33	2.51
Waiting time or delay (e.g., for a bus or train)	2.51	2.41
Carelessness or errors due to lack of attention	2.37	2.91

Gossip or gossip from others (including on social media)	2.1	2.8
Discrimination or bullying by another person (including on social media)	1.79	2.73
Nightmares	1.8	3.08
Commuting to work/training/school/university	3.85	2.14
Minor legal violation (e.g., fine for an offense)	1.29	2.51
Inconvenience with authority, office, or other institution (e.g., tax office, bank, company)	1.61	3.2
Conflict or disagreement in the workplace (e.g., with superiors or colleagues)	1.72	2.96
Conflict or disagreement with close persons (e.g., parents, siblings, partner)	2.14	3.47
Conflict or disagreement between close persons (e.g., between parents, between friends)	2.13	3.29
Conflict or disagreement with other non-close persons (e.g., bus driver, neighbor)	1.57	2.57
Conflict or disagreement with your child/children	1	1.36
Problem with childcare	1.08	1.36
Errands or driving service for others	1.99	1.9
Problem or inconvenience because your friends or relatives live too far away	2.78	3.24
Problem due to lack of support or help from others	2.28	3.42
Problem with your pet (e.g., illness, unwanted behavior)	2.27	2.82
Impairment due to unsafe environment (e.g., unsafe living environment)	2.29	2.62
Impairment due to dirt, pollution, or odor (e.g., in the neighborhood or apartment)	2.5	2.84
Problem due to insufficient money (e.g., for basic needs, emergencies, or special desires)	2.58	3.1
Others owe you money	2.19	1.88
You owe money to others	2.18	2.42
High or unexpected financial burden (e.g., buying expensive products or items, car repair costs)	1.64	3.1
Financial matter (e.g., paying bills, dealing with financial planning for retirement)	2.31	2.64
Unexpected or unwanted visit	1.45	2.2
Side effects of medication	2.28	3
Personal physical complaint (e.g., minor illness or pain)	3.01	3.23
Physical complaint of a close person	2.87	3
Lack of sleep or sleep problems	3	3.31
Doctor's visit	1.42	2.2
Home office or paperwork (e.g., filling out forms)	2.98	2.67
Household management (e.g., cooking, cleaning, or shopping)	4.51	2.13
Making a minor repair (e.g., in your own home)	1.69	1.85

Problem with a technical device (e.g., computer, household appliance, electronic device)	2.12	2.96
Maintenance or upkeep of an item (e.g., of the car)	1.65	2.39
Unpleasant or bad weather (e.g., rain, heat, cold)	3.01	2.49
Disruptive behavior or misconduct of others (e.g., inconsiderate smokers, annoying neighbors)	2.19	3.08
Bad food (e.g., in the cafeteria or canteen)	1.8	2.44
Noise (e.g., street noise, aircraft noise)	3.12	2.72
Traffic jam	1.95	2.79
Looking for a parking space	2.16	2.49
Problem with a means of communication (e.g., internet, phone)	2.57	3.05
Performance situation at work/school/university (e.g., exam)	2.92	3.59
High performance demand or workload at work/school/university	4.37	3.7
Boring activity (e.g., at work or in studies)	2.75	2.66
Meeting (e.g., at work, in studies, in the club)	2.27	2.08
Unregulated or too long working hours	3.34	2.96
Problem with planning or scheduling appointments	2.36	3.1
Time pressure	3.43	3.5
Bad news (e.g., rejection of application, notification of poor exam results)	1.69	3.7
Problem due to searching for an education/training/workplace	2.61	3.39
Problem due to apartment search or moving	2.86	3.5

Supplementary Table 3. MARP: Prediction of stressor reactivity (SR) by PASS-content and PASS-process in the most stressor-exposed participants (top two terciles of mean E between B0 and B2), controlling for baseline (B0) covariates.

Predictor time point (battery)	B0						B1			
Outcome interval (SR score)	B0-B2 (~3.7 yrs)		B0-B1 (~1.9 yrs)		3 monitorings post B0 (~9 m)		B1-B2 (~1.6 yrs)		3 monitorings post B1 (~9 m)	
	PASS-content	PASS-process	PASS-content	PASS-process	PASS-content	PASS-process	PASS-content	PASS-process	PASS-content	PASS-process
PAS	**-0.215**	-0.139	**-0.220**	-0.105	-0.124	-0.117	**-0.394**	-0.287	**-0.393**	-0.273
	**(-0.417, -0.013)**	(-0.343, 0.066)	**(-0.428, -0.013)**	(-0.317, 0.106)	(-0.328, 0.080)	(-0.325, 0.092)	**(-0.657, -0.131)**	(-0.577, 0.003)	**(-0.635, -0.151)**	(-0.546, 0.0001)
	**p = 0.041**	p = 0.188	**p = 0.041**	p = 0.333	p = 0.237	p = 0.276	**p = 0.005**	p = 0.059	**p = 0.003**	p = 0.056
Age	-0.207	-0.230	-0.211	-0.227	-0.039	-0.037	-0.184	-0.128	-0.148	-0.098
	(-0.449, 0.036)	(-0.479, 0.020)	(-0.460, 0.039)	(-0.486, 0.033)	(-0.285, 0.208)	(-0.285, 0.210)	(-0.525, 0.157)	(-0.494, 0.237)	(-0.467, 0.170)	(-0.443, 0.247)
	p = 0.099	p = 0.075	p = 0.102	p = 0.091	p = 0.760	p = 0.769	p = 0.295	p = 0.495	p = 0.366	p = 0.579
Sex	0.383	0.323	0.360	0.324	0.150	0.103	0.057	0.130	0.119	0.140
	(-0.031, 0.796)	(-0.099, 0.745)	(-0.063, 0.783)	(-0.115, 0.762)	(-0.262, 0.562)	(-0.309, 0.515)	(-0.508, 0.621)	(-0.472, 0.733)	(-0.387, 0.626)	(-0.407, 0.686)
	p = 0.074	p = 0.138	p = 0.100	p = 0.153	p = 0.478	p = 0.625	p = 0.845	p = 0.674	p = 0.647	p = 0.619
Childhood trauma	0.073	0.222	0.097	0.230	-0.097	-0.016	0.005	-0.033	0.374	0.321
	(-0.475, 0.621)	(-0.327, 0.771)	(-0.464, 0.659)	(-0.345, 0.804)	(-0.698, 0.505)	(-0.618, 0.586)	(-0.805, 0.815)	(-0.896, 0.830)	(-0.366, 1.115)	(-0.475, 1.117)
	p = 0.795	p = 0.431	p = 0.736	p = 0.436	p = 0.754	p = 0.960	p = 0.990	p = 0.941	p = 0.326	p = 0.433
Smoking	0.065	0.069	0.067	0.074	0.070	0.074	0.013	0.050	-0.002	0.037
	(-0.029, 0.160)	(-0.026, 0.164)	(-0.029, 0.163)	(-0.025, 0.173)	(-0.024, 0.164)	(-0.020, 0.167)	(-0.113, 0.138)	(-0.080, 0.181)	(-0.119, 0.115)	(-0.086, 0.159)
	p = 0.177	p = 0.160	p = 0.176	p = 0.146	p = 0.152	p = 0.128	p = 0.844	p = 0.455	p = 0.973	p = 0.562
Number of assessments	**-0.181**	**-0.233**	0.038	0.040			-0.159	-0.169		
	**(-0.348, -0.014)**	**(-0.407, -0.060)**	(-0.300, 0.376)	(-0.317, 0.396)			(-0.388, 0.071)	(-0.412, 0.074)		
	**p = 0.037**	**p = 0.011**	p = 0.826	p = 0.829			p = 0.181	p = 0.180		
Constant	5.504	**6.519**	2.964	3.153	0.526	0.450	4.544	3.477	2.240	1.315
	(0.098, 10.911)	**(0.944, 12.093)**	(-2.710, 8.639)	(-2.691, 8.997)	(-4.370, 5.422)	(-4.464, 5.364)	(-2.570, 11.657)	(-4.131, 11.085)	(-4.081, 8.560)	(-5.531, 8.161)
	p = 0.050	**p = 0.025**	p = 0.309	p = 0.294	p = 0.834	p = 0.859	p = 0.216	p = 0.375	p = 0.491	p = 0.709
Observations (n)	**87**	**85**	**87**	85	74	73	61	59	**62**	60
R^2	**0.198**	**0.190**	**0.147**	0.115	0.066	0.063	0.190	0.123	**0.186**	0.103
Adjusted R^2	**0.138**	**0.127**	**0.083**	0.047	-0.003	-0.007	0.100	0.021	**0.114**	0.020
Residual Std. Error	**0.925 (df = 80)**	**0.936 (df = 78)**	**0.946 (df = 80)**	0.974 (df = 78)	0.856 (df = 68)	0.857 (df = 67)	1.043 (df = 54)	1.104 (df = 52)	**0.970 (df = 56)**	1.036 (df = 54)
F Statistic	**3.294 (df = 6; 80)**	**3.045 (df = 6; 78)**	**2.289 (df = 6; 80)**	1.689 (df = 6; 78)	0.960 (df = 5; 68)	0.904 (df = 5; 67)	2.110 (df = 6; 54)	1.211 (df = 6; 52)	**2.568 (df = 5; 56)**	1.236 (df = 5; 54)
F Statistic (p-value)	**0.006**	**0.01**	**0.043**	0.135	0.448	0.484	0.067	0.316	**0.037**	0.305

Note: Results of linear regression models, not adjusted for multiple comparisons. Estimates are standardized betas; 95% Confidence Interval reported in brackets. Values in bold are statistically significant at a level $p < 0.05$ (two-sided)

Supplementary Table 4. MARP: Prediction of stressor reactivity (SR) by perceived social support and perceived good stress recovery, controlling for baseline (B0) covariates.

Predictor time point (battery)	B0						B1			
Outcome interval (SR score)	B0-B2 (~3.7 yrs)		B0-B1 (~1.9 yrs)		3 monitorings post B0 (~9 m)		B1-B2 (~1.6 yrs)		3 monitorings post B1 (~9 m)	
	Social Support	Stress recovery	Social Support	Stress recovery	Social Support	Stress recovery	Social Support	Stress recovery	Social Support	Stress recovery
Resilience Factor	**-0.195**<br>**(-0.384, -0.006)**<br>**p = 0.046**	**-0.250**<br>**(-0.403, -0.096)**<br>**p = 0.002**	-0.182<br>(-0.380, 0.016)<br>p = 0.074	**-0.313**<br>**(-0.474, -0.152)**<br>**p = 0.0003**	0.130<br>(-0.111, 0.371)<br>p = 0.294	**-0.186**<br>**(-0.345, -0.028)**<br>**p = 0.024**	**-0.464**<br>**(-0.752, -0.177)**<br>**p = 0.003**	**-0.324**<br>**(-0.539, -0.110)**<br>**p = 0.004**	**-0.316**<br>**(-0.595, -0.037)**<br>**p = 0.030**	**-0.374**<br>**(-0.578, -0.168)**<br>**p = 0.001**
Age	-0.130<br>(-0.328, 0.067)<br>p = 0.198	-0.105<br>(-0.298, 0.088)<br>p = 0.289	-0.125<br>(-0.337, 0.086)<br>p = 0.249	-0.101<br>(-0.303, 0.102)<br>p = 0.332	-0.053<br>(-0.274, 0.168)<br>p = 0.638	-0.056<br>(-0.273, 0.161)<br>p = 0.613	-0.013<br>(-0.280, 0.254)<br>p = 0.924	-0.118<br>(-0.382, 0.145)<br>p = 0.382	-0.001<br>(-0.255, 0.254)<br>p = 0.995	-0.104<br>(-0.343, 0.136)<br>p = 0.400
Sex	**0.440**<br>**(0.109, 0.770)**<br>**p = 0.011**	**0.342**<br>**(0.017, 0.666)**<br>**p = 0.042**	**0.391**<br>**(0.036, 0.747)**<br>**p = 0.034**	0.285<br>(-0.056, 0.626)<br>p = 0.104	0.254<br>(-0.101, 0.608)<br>p = 0.164	0.232<br>(-0.115, 0.580)<br>p = 0.194	0.395<br>(-0.047, 0.837)<br>p = 0.084	0.337<br>(-0.097, 0.770)<br>p = 0.133	0.303<br>(-0.105, 0.711)<br>p = 0.150	0.239<br>(-0.143, 0.621)<br>p = 0.225
Childhood trauma	0.126<br>(-0.424, 0.675)<br>p = 0.655	0.376<br>(-0.091, 0.844)<br>p = 0.118	0.180<br>(-0.397, 0.757)<br>p = 0.544	0.410<br>(-0.073, 0.894)<br>p = 0.099	0.307<br>(-0.349, 0.964)<br>p = 0.361	0.143<br>(-0.425, 0.712)<br>p = 0.622	0.282<br>(-0.309, 0.873)<br>p = 0.353	0.281<br>(-0.308, 0.869)<br>p = 0.353	0.367<br>(-0.192, 0.927)<br>p = 0.202	0.331<br>(-0.200, 0.863)<br>p = 0.226
Smoking	0.035<br>(-0.051, 0.120)<br>p = 0.429	0.048<br>(-0.034, 0.129)<br>p = 0.254	0.037<br>(-0.053, 0.126)<br>p = 0.426	0.047<br>(-0.037, 0.131)<br>p = 0.279	0.065<br>(-0.029, 0.160)<br>p = 0.176	0.044<br>(-0.044, 0.131)<br>p = 0.330	-0.012<br>(-0.116, 0.092)<br>p = 0.825	0.049<br>(-0.049, 0.147)<br>p = 0.327	0.005<br>(-0.095, 0.104)<br>p = 0.924	0.046<br>(-0.043, 0.135)<br>p = 0.312
Number of assessments	**-0.158**<br>**(-0.298, -0.018)**<br>**p = 0.030**	**-0.156**<br>**(-0.293, -0.019)**<br>**p = 0.028**	0.070<br>(-0.201, 0.341)<br>p = 0.615	0.104<br>(-0.157, 0.364)<br>p = 0.436			-0.055<br>(-0.229, 0.119)<br>p = 0.537	-0.032<br>(-0.206, 0.143)<br>p = 0.722		
Constant	3.602<br>(-0.817, 8.021)<br>p = 0.113	2.902<br>(-1.402, 7.206)<br>p = 0.189	1.014<br>(-3.567, 5.596)<br>p = 0.666	0.168<br>(-4.199, 4.535)<br>p = 0.940	0.136<br>(-4.280, 4.551)<br>p = 0.953	0.450<br>(-3.874, 4.774)<br>p = 0.839	-0.329<br>(-5.905, 5.248)<br>p = 0.909	1.522<br>(-3.917, 6.960)<br>p = 0.585	-0.931<br>(-6.020, 4.157)<br>p = 0.721	1.125<br>(-3.663, 5.913)<br>p = 0.647
Observations (n)	**133**	**133**	**128**	**128**	114	114	**84**	**86**	86	**88**
R^2	**0.162**	**0.200**	**0.106**	**0.180**	0.050	0.085	**0.172**	**0.164**	0.113	**0.186**
Adjusted R^2	**0.122**	**0.162**	**0.062**	**0.140**	0.006	0.043	**0.107**	**0.101**	0.057	**0.136**
Residual Std. Error	**0.934 (df = 126)**	**0.913 (df = 126)**	**0.982 (df = 121)**	**0.940 (df = 121)**	0.939 (df = 108)	0.922 (df = 108)	**0.976 (df = 77)**	**0.978 (df = 79)**	0.934 (df = 80)	**0.891 (df = 82)**
F Statistic	**4.071 (df = 6; 126)**	**5.237 (df = 6; 126)**	**2.399 (df = 6; 121)**	**4.436 (df = 6; 121)**	1.144 (df = 5; 108)	2.014 (df = 5; 108)	**2.661 (df = 6; 77)**	**2.588 (df = 6; 79)**	2.036 (df = 5; 80)	**3.748 (df = 5; 82)**
F Statistic (p-value)	**<0.001**	**<0.001**	**0.0317**	**<0.001**	0.342	0.0824	**0.0212**	**0.0243**	0.0824	**0.00417**

Note: Results of linear regression models, not adjusted for multiple comparisons. Estimates are standardized betas; 95% Confidence Interval reported in brackets. Values in bold are statistically significant at a level p < 0.05 (two-sided)

Supplementary Table 5. MARP: Prediction of stressor reactivity (SR) by perceived social support and perceived good stress recovery in the most stressor-exposed participants (top two terciles of mean E between B0 and B2), controlling for baseline (B0) covariates.

Predictor time point (battery)	B0						B1			
Outcome interval (SR score)	B0-B2 (~3.7 yrs)		B0-B1 (~1.9 yrs)		3 monitorings post B0 (~9 m)		B0-B2 (~3.7 yrs)		B0-B1 (~1.9 yrs)	
	Social Support	Stress recovery	Social Support	Stress recovery	Social Support	Stress recovery	Social Support	Stress recovery	Social Support	Stress recovery
Resilience Factor	-0.227	**-0.279**	-0.221	**-0.335**	0.116	-0.161	**-0.393**	**-0.457**	**-0.314**	**-0.544**
	(-0.461, 0.007)	**(-0.473, -0.085)**	(-0.462, 0.020)	**(-0.531, -0.140)**	(-0.118, 0.351)	(-0.352, 0.030)	**(-0.683, -0.104)**	**(-0.702, -0.212)**	**(-0.596, -0.032)**	**(-0.784, -0.303)**
	p = 0.061	**p = 0.007**	p = 0.077	**p = 0.002**	p = 0.335	p = 0.104	**p = 0.011**	**p = 0.001**	**p = 0.034**	**p = 0.00005**
Age	-0.206	-0.152	-0.209	-0.144	-0.032	-0.018	-0.131	-0.226	-0.099	-0.214
	(-0.449, 0.038)	(-0.392, 0.088)	(-0.460, 0.042)	(-0.386, 0.098)	(-0.279, 0.215)	(-0.263, 0.226)	(-0.475, 0.214)	(-0.542, 0.089)	(-0.432, 0.233)	(-0.501, 0.074)
	p = 0.103	p = 0.217	p = 0.106	p = 0.248	p = 0.800	p = 0.885	p = 0.462	p = 0.166	p = 0.561	p = 0.152
Sex	0.360	0.313	0.338	0.281	0.134	0.092	0.083	0.037	0.148	0.074
	(-0.054, 0.775)	(-0.093, 0.718)	(-0.087, 0.763)	(-0.126, 0.688)	(-0.278, 0.546)	(-0.316, 0.500)	(-0.506, 0.672)	(-0.500, 0.574)	(-0.396, 0.691)	(-0.394, 0.542)
	p = 0.093	p = 0.135	p = 0.124	p = 0.181	p = 0.525	p = 0.661	p = 0.784	p = 0.893	p = 0.597	p = 0.758
Childhood trauma	-0.057	0.200	-0.024	0.238	0.079	-0.015	0.199	0.169	0.437	0.394
	(-0.649, 0.535)	(-0.330, 0.729)	(-0.630, 0.583)	(-0.295, 0.771)	(-0.584, 0.741)	(-0.611, 0.580)	(-0.482, 0.879)	(-0.471, 0.808)	(-0.212, 1.086)	(-0.181, 0.969)
	p = 0.852	p = 0.463	p = 0.940	p = 0.385	p = 0.817	p = 0.961	p = 0.570	p = 0.608	p = 0.193	p = 0.185
Smoking	0.044	0.070	0.047	0.071	0.096	0.074	-0.012	0.049	-0.012	0.038
	(-0.055, 0.144)	(-0.021, 0.162)	(-0.055, 0.150)	(-0.020, 0.163)	(-0.004, 0.196)	(-0.019, 0.166)	(-0.139, 0.115)	(-0.067, 0.164)	(-0.134, 0.111)	(-0.067, 0.142)
	p = 0.386	p = 0.137	p = 0.367	p = 0.131	p = 0.066	p = 0.124	p = 0.855	p = 0.416	p = 0.852	p = 0.482
Number of assessments	**-0.187**	**-0.195**	0.030	0.049			-0.131	-0.135		
	**(-0.355, -0.020)**	**(-0.359, -0.032)**	(-0.309, 0.370)	(-0.275, 0.373)			(-0.356, 0.094)	(-0.344, 0.074)		
	**p= 0.032**	**p= 0.022**	p= 0.863	p= 0.769			p = 0.258	p = 0.211		
Constant	**5.779**	4.595	3.199	1.562	0.184	0.122	3.107	5.018	1.210	3.541
	**(0.340, 11.218)**	(-0.734, 9.925)	(-2.513, 8.911)	(-3.964, 7.087)	(-4.737, 5.105)	(-4.731, 4.976)	(-4.187, 10.402)	(-1.544, 11.580)	(-5.481, 7.901)	(-2.221, 9.302)
	**p = 0.041**	p = 0.095	p = 0.276	p = 0.582	p = 0.942	p = 0.961	p = 0.408	p = 0.140	p = 0.725	p = 0.234
Observations (n)	**87**	**87**	87	**87**	74	74	61	**63**	62	**64**
R^2	**0.191**	**0.231**	0.135	**0.212**	0.059	0.083	0.171	**0.251**	0.125	**0.296**
Adjusted R^2	**0.131**	**0.173**	0.070	**0.152**	-0.010	0.016	0.079	**0.171**	0.047	**0.235**
Residual Std. Error	**0.929 (df = 80)**	**0.906 (df = 80)**	0.952 (df = 80)	**0.909 (df = 80)**	0.859 (df = 68)	0.848 (df = 68)	1.049 (df = 54)	**0.995 (df = 56)**	1.008 (df = 56)	**0.900 (df = 58)**
F Statistic	**3.152 (df = 6; 80)**	**4.002 (df = 6; 80)**	2.084 (df = 6; 80)	**3.577 (df = 6; 80)**	0.860 (df = 5; 68)	1.233 (df = 5; 68)	1.858 (df = 6; 54)	**3.130 (df = 6; 56)**	1.606 (df = 5; 56)	**4.879 (df = 5; 58)**
F Statistic (p-value)	**0.008**	**0.001**	0.064	**0.003**	0.512	0.303	0.105	**0.01**	0.173	**<0.001**

Note: Results of linear regression models, not adjusted for multiple comparisons. Estimates are standardized betas; 95% Confidence Interval reported in brackets. Values in bold are statistically significant at a level p < 0.05 (two-sided). Results for PASS-process are shown for descriptive purposes only

Supplementary Table 16. MARP: Results of covariate selection.

Variables	p value	In model p<0.2
age in years	0.456	Yes*
sex	0.010	Yes*
relationship status	0.395	No
employment status	0.292	No
education	0.242	No
student status	0.368	No
mental illnesses in the family	0.652	No
monthly income	0.992	No
monthly household income	0.804	No
smoking[1]	0.091	Yes
cannabis[2]	0.329	No
life events score[3]	0.570	No
childhood trauma[4]	0.025	Yes
number of assessments[5]	0.002	Yes

Note: [1] Number of cigarettes per day/month; [2] Times per month; [3] Summary of life events score; [4] Childhood trauma questionnaire (CTQ); [5] Number of assessments between the respective baselines. *were included independent of p value.

Observational replication sample: LORA

Supplementary Table 6. LORA: demographics.

Variables		Mean	St. Dev.	Min	Max	n	n in %
age in years		28.84	8.04	18	50		
sex	female					686	66.3%
	male					348	33.7%
relationship status	single					824	80.0%
	married					178	17.3%
	living apart					9	0.9%
	divorced/separated					17	1.7%
	widowed					2	0.2%
employment status	unemployed (NOT due to health reasons)					24	2.3%
	unemployed (for health reasons)					2	0.2%
	working part-time (NOT for health reasons)					130	12.6%
	working part-time (for health reasons)					3	0.3%
	working full-time					330	32.0%
	pupil, student or in training					542	52.6%
education	no qualification					1	0.1%
	secondary school leaving certificate (low)					2	0.2%
	secondary school leaving certificate (high)					25	2.4%
	A-levels					396	38.3%
	completed vocational training					144	13.9%
	completed university					466	45.1%
persons household income[1]		1.91	0.94	0	8		
household income[2]	Refused to answer					113	10.9%
	below 800 euros					118	11.4%
	801 to 1500 euros					150	14.5%
	1501 to 2000 euros					111	10.7%
	2001 to 3000 euros					175	16.9%
	3001 to 5000 euros					233	22.5%
	more than 5000 euros					134	13.0%
smoking	yes					128	12.4%
	no					906	87.6%
life events score[3]		12.25	7.43	0	53		
childhood trauma[4]		33.43	10.04	25	102		
alcohol use[5]		3.84	2.78	0	20		

Note: [1] number of persons contributing to your household income; [2] average monthly net income of your household; [3] Summary of life events score before baseline; [4] Childhood trauma questionnaire (CTQ); [5] Alcohol use disorder identification test (AUDIT) . St. Dev = standard deviation, Min = minimum, Max = maximum.

Supplementary Table 7a. LORA: Life events (LE) exposure in the analyzed sample from B0 to B2.

Variables	Average report (Mean)	Average severity (Mean)
Lost job	0.02	1.87
Traumatic incident at work	0.06	2.32
Wedding planning	0.07	1.36
Bought or sold a house, moving house	0.15	2
Major house renovation	0.09	1.83
Had something stolen or vanished	0.01	1.41
Legal problems	0.05	1.16
Serious financial problems	0.1	2.25
Serious illness, accident or diagnosis of oneself or a close family member (child, parent, sibling, grandparent)	0.18	2.47
Serious illness, accident or diagnosis of a close friend or partner	0.07	2.15
Death of a family member	0.05	2.42
Death of a friend (other than boyfriend/girlfriend)	0.02	2.42
Death of a beloved pet	0.02	2.61
Parents separated	0.01	2.17
Constant arguments between family members	0.22	2.06
Broke up with boyfriend/girlfriend or spouse	0.06	2.86
Serious arguments with boyfriend/girlfriend or spouse	0.2	2.63
Serious problems in relationships with friends	0.14	2.12
Child started school	0.01	1.18
Increased care for elderly or ill person	0.06	1.9
You/a partner had an abortion	0	2.32
Serious physical illness – unable to work or carry out normal activities	0.06	2.29
Injury – unable to work or carry out normal activities	0.06	2.08
You/a partner had a difficult pregnancy or miscarriage	0.01	2.83
Been physically assaulted or mugged	0	2.24
Been sexually assaulted	0	1.62
Other impactful event (e.g. exam, car accident, house fire, earthquake, war)	0.27	2.38

Supplementary Table 7b. LORA: Daily hassles (DH) exposure in the analyzed sample from B0 to B2.

Variables	Average days (Mean)	Average severity (Mean)
Losing or misplacing items	2.15	0.86
Negative event in the media	3.85	1.07
Negative political event	3.23	0.96
Social obligation	3.06	0.82
Interruption in activity (e.g., work or leisure)	3.55	1.02

Waiting time or delay (e.g., for a bus or train)	2.57	0.9
Carelessness or errors due to lack of attention	2.25	1.13
Gossip or gossip from others (including on social media)	2.49	0.87
Discrimination or bullying by another person (including on social media)	2.17	0.57
Nightmares	1.86	1.14
Commuting to work/training/school/university	4.12	0.65
Minor legal violation (e.g., fine for an offense)	1.25	0.45
Inconvenience with authority, office, or other institution (e.g., tax office, bank, company)	1.66	1.12
Conflict or disagreement in the workplace (e.g., with superiors or colleagues)	1.98	1.22
Conflict or disagreement with close persons (e.g., parents, siblings, partner)	2.1	1.65
Conflict or disagreement between close persons (e.g., between parents, between friends)	2.1	1.19
Conflict or disagreement with other non-close persons (e.g., bus driver, neighbor)	1.66	0.72
Conflict or disagreement with your child/children	3.67	0.88
Problem with childcare	2.88	0.61
Errands or driving service for others	1.94	0.33
Problem or inconvenience because your friends or relatives live too far away	2.77	1.28
Problem due to lack of support or help from others	2.54	1.24
Problem with your pet (e.g., illness, unwanted behavior)	2.84	0.73
Impairment due to unsafe environment (e.g., unsafe living environment)	2.64	0.52
Impairment due to dirt, pollution, or odor (e.g., in the neighborhood or apartment)	2.75	1.01
Problem due to insufficient money (e.g., for basic needs, emergencies, or special desires)	3.39	1.23
Others owe you money	3.3	0.34
You owe money to others	3.2	0.6
High or unexpected financial burden (e.g., buying expensive products or items, car repair costs)	1.76	1.13
Financial matter (e.g., paying bills, dealing with financial planning for retirement)	2.29	0.8
Unexpected or unwanted visit	1.56	0.49
Side effects of medication	3.2	0.61
Personal physical complaint (e.g., minor illness or pain)	3.31	1.38
Physical complaint of a close person	3.22	1.07
Lack of sleep or sleep problems	3.17	1.48
Doctor's visit	1.42	0.54
Home office or paperwork (e.g., filling out forms)	2.67	0.93

Household management (e.g., cooking, cleaning, or shopping)	4.82	0.76
Making a minor repair (e.g., in your own home)	1.78	0.39
Problem with a technical device (e.g., computer, household appliance, electronic device)	1.99	1
Maintenance or upkeep of an item (e.g., of the car)	1.46	0.43
Unpleasant or bad weather (e.g., rain, heat, cold)	3.07	0.77
Disruptive behavior or misconduct of others (e.g., inconsiderate smokers, annoying neighbors)	2.51	1.2
Bad food (e.g., in the cafeteria or canteen)	2.08	0.65
Noise (e.g., street noise, aircraft noise)	3.61	0.94
Traffic jam	2.05	0.94
Looking for a parking space	2.46	0.71
Problem with a means of communication (e.g., internet, phone)	2.4	0.93
Performance situation at work/school/university (e.g., exam)	3.18	1.51
High performance demand or workload at work/school/university	3.98	1.62
Boring activity (e.g., at work or in studies)	2.67	0.98
Meeting (e.g., at work, in studies, in the club)	2.56	0.59
Unregulated or too long working hours	3.35	1.09
Problem with planning or scheduling appointments	2.25	1.15
Time pressure	3.4	1.55
Bad news (e.g., rejection of application, notification of poor exam results)	1.71	1.25
Problem due to searching for an education/training/workplace	2.74	0.82
Problem due to apartment search or moving	3.1	0.79

Supplementary Table 8. LORA: Prediction of stressor reactivity (SR) by perceived social support and perceived good stress recovery, controlling for baseline B0) covariates.

Predictor time point (battery)	B0						B1			
Outcome interval (SR score)	B0-B2 (~3.7 yrs)		B0-B1 (~1.9 yrs)		3 monitorings post B0 (~9 m)		B1-B2 (~1.6 yrs)		3 monitorings post B1 (~9 m)	
	Social Support	Stress recovery	Social Support	Stress recovery	Social Support	Stress recovery	Social Support	Stress recovery	Social Support	Stress recovery
Resilience Factor	**-0.111**<br>**(-0.161, -0.062)**<br>**p = 0.00002**	**-0.201**<br>**(-0.246, -0.156)**<br>**p < 0.00001**	**-0.106**<br>**(-0.159, -0.054)**<br>**p = 0.0001**	**-0.223**<br>**(-0.270, -0.175)**<br>**p < 0.00001**	**-0.134**<br>**(-0.196, -0.071)**<br>**p = 0.00004**	**-0.254**<br>**(-0.310, -0.197)**<br>**p < 0.00001**	**-0.233**<br>**(-0.294, -0.171)**<br>**p < 0.00001**	**0.146**<br>**(-0.215, -0.077)**<br>**p = 0.00004**	**-0.232**<br>**(-0.295, -0.169)**<br>**p < 0.00001**	**-0.111**<br>**(-0.184, -0.037)**<br>**p = 0.004**
Age	-0.002<br>(-0.008, 0.005)<br>p = 0.601	-0.0002<br>(-0.006, 0.006)<br>p = 0.954	-0.004<br>(-0.010, 0.003)<br>p = 0.286	-0.002<br>(-0.009, 0.004)<br>p = 0.491	-0.003<br>(-0.011, 0.004)<br>p = 0.393	-0.002<br>(-0.009, 0.006)<br>p = 0.691	0.003<br>(-0.005, 0.011)<br>p = 0.439	0.002<br>(-0.006, 0.011)<br>p = 0.557	0.001<br>(-0.007, 0.010)<br>p = 0.744	0.002<br>(-0.006, 0.011)<br>p = 0.621
Sex	**0.336**<br>**(0.239, 0.433)**<br>**p = 0.000**	**0.246**<br>**(0.153, 0.340)**<br>**p = 0.00000**	**0.358**<br>**(0.255, 0.461)**<br>**p = 0.000**	**0.264**<br>**(0.165, 0.364)**<br>**p = 0.00000**	**0.341**<br>**(0.218, 0.465)**<br>**p = 0.00000**	**0.228**<br>**(0.109, 0.348)**<br>**p = 0.0002**	**0.203**<br>**(0.072, 0.334)**<br>**p = 0.003**	**0.299**<br>**(0.164, 0.434)**<br>**p = 0.00002**	**0.191**<br>**(0.054, 0.327)**<br>**p = 0.007**	**0.287**<br>**(0.146, 0.429)**<br>**p = 0.0001**
Childhood trauma	0.004<br>(-0.001, 0.008)<br>p = 0.150	0.004<br>(-0.0002, 0.009)<br>p = 0.064	**0.005**<br>**(0.0004, 0.011)**<br>**p = 0.036**	**0.006**<br>**(0.001, 0.010)**<br>**p = 0.018**	**0.007**<br>**(0.001, 0.012)**<br>**p = 0.031**	**0.007**<br>**(0.002, 0.013)**<br>**p = 0.009**	0.004<br>(-0.003, 0.010)<br>p = 0.260	0.002<br>(-0.005, 0.008)<br>p = 0.583	0.005<br>(-0.002, 0.012)<br>p = 0.130	0.005<br>(-0.002, 0.012)<br>p = 0.171
Income	**-0.036**<br>**(-0.061, -0.011)**<br>**p = 0.006**	**-0.029**<br>**(-0.054, -0.004)**<br>**p = 0.022**	**-0.033**<br>**(-0.061, -0.006)**<br>**p = 0.016**	-0.026<br>(-0.052, 0.001)<br>p = 0.057	**-0.038**<br>**(-0.070, -0.006)**<br>**p = 0.022**	-0.028<br>(-0.060, 0.004)<br>p = 0.082	**-0.045**<br>**(-0.080, -0.009)**<br>**p = 0.015**	**-0.051**<br>**(-0.087, -0.015)**<br>**p = 0.006**	**-0.057**<br>**(-0.094, -0.020)**<br>**p = 0.003**	**-0.067**<br>**(-0.104, -0.029)**<br>**p = 0.001**
Constant	**-0.477**<br>**(-0.758, -0.197)**<br>**p = 0.001**	**-0.424**<br>**(-0.697, -0.151)**<br>**p = 0.003**	**-0.543**<br>**(-0.843, -0.244)**<br>**p = 0.0004**	**-0.469**<br>**(-0.758, -0.179)**<br>**p = 0.002**	**-0.527**<br>**(-0.880, -0.173)**<br>**p = 0.004**	**-0.462**<br>**(-0.805, -0.120)**<br>**p = 0.009**	-0.366<br>(-0.748, 0.016)<br>p = 0.061	**-0.414**<br>**(-0.802, -0.025)**<br>**p = 0.038**	-0.292<br>(-0.698, 0.114)<br>p = 0.160	**-0.425**<br>**(-0.843, -0.008)**<br>**p = 0.047**
Observations (n)	**1,039**	**1,040**	**1,039**	**1,040**	**933**	**934**	**754**	**759**	**705**	**707**
R^2	**0.071**	**0.119**	**0.071**	**0.127**	**0.062**	**0.118**	**0.104**	**0.057**	**0.111**	**0.059**
Adjusted R^2	**0.066**	**0.114**	**0.066**	**0.123**	**0.057**	**0.113**	**0.098**	**0.051**	**0.104**	**0.052**
Residual Std. Error	**0.742 (df = 1033)**	**0.723 (df = 1034)**	**0.791 (df = 1033)**	**0.767 (df = 1034)**	**0.889 (df = 927)**	**0.864 (df = 928)**	**0.849 (df = 748)**	**0.871 (df = 753)**	**0.856 (df = 699)**	**0.881 (df = 701)**
F Statistic	**15.718 (df = 5; 1033)**	**27.831(df = 5; 1034)**	**15.698 (df = 5; 1033)**	**30.206 (df = 5; 1034)**	**12.308 (df = 5; 927)**	**24.742 (df = 5; 928)**	**17.323 (df = 5; 748)**	**9.130 (df = 5; 753)**	**17.418 (df = 5; 699)**	**8.750 (df = 5; 701)**
F Statistic (p-value)	**<0.001**	**<0.001**	**<0.001**	**<0.001**	**<0.001**	**<0.001**	**<0.001**	**<0.001**	**<0.001**	**<0.001**

Note: Results of linear regression models, not adjusted for multiple comparisons. Estimates are standardized betas; 95% Confidence Interval reported in brackets. Values in bold are statistically significant at a level p < 0.05 (two-sided).

Supplementary Table 9. LORA: Prediction of stressor reactivity (SR) by PASS-content and PASS-process in the most stressor-exposed participants (top two terciles of mean E between B0 and B2), controlling for baseline (B0) covariates.

Predictor time point (battery)	B0						B1			
Outcome interval (SR score)	B0-B2 (~3.7 yrs)		B0-B1 (~1.9 yrs)		3 monitorings post B0 (~9 m)		B1-B2 (~1.6 yrs)		3 monitorings post B1 (~9 m)	
	PASS-content	PASS-process	PASS-content	PASS-process	PASS-content	PASS-process	PASS-content	PASS-process	PASS-content	PASS-process
PAS	**-0.242**	**0.137**	**-0.255**	**-0.150**	**-0.226**	**-0.131**	**-0.252**	**-0.111**	**-0.249**	-0.067
	**(-0.300, -0.185)**	**(-0.196, -0.078)**	**(-0.315, -0.194)**	**(-0.213, -0.088)**	**(-0.300, -0.152)**	**(-0.206, -0.057)**	**(-0.332, -0.172)**	**(-0.192, -0.030)**	**(-0.332, -0.165)**	(-0.152, 0.017)
	**p < 0.00001**	**p = 0.00001**	**p < 0.00001**	**p = 0.00001**	**p < 0.00001**	**p = 0.001**	**p < 0.00001**	**p = 0.008**	**p < 0.00001**	p = 0.118
Age	-0.0001	-0.0004	-0.003	-0.003	0.00002	-0.0003	0.004	0.006	0.001	0.003
	(-0.008, 0.008)	(-0.008, 0.008)	(-0.011, 0.005)	(-0.012, 0.005)	(-0.010, 0.010)	(-0.010, 0.010)	(-0.006, 0.015)	(-0.005, 0.016)	(-0.010, 0.012)	(-0.008, 0.014)
	p = 0.983	p = 0.931	p = 0.492	p = 0.452	p = 0.998	p = 0.962	p = 0.404	p = 0.308	p = 0.820	p = 0.607
Sex	**0.379**	**0.342**	**0.393**	**0.353**	**0.357**	**0.324**	**0.298**	**0.290**	**0.274**	**0.287**
	**(0.258, 0.501)**	**(0.215, 0.468)**	**(0.264, 0.522)**	**(0.220, 0.487)**	**(0.200, 0.514)**	**(0.163, 0.484)**	**(0.126, 0.470)**	**(0.114, 0.466)**	**(0.095, 0.452)**	**(0.103, 0.471)**
	**p = 0.000**	**p = 0.00000**	**p = 0.000**	**p = 0.00000**	**p = 0.00001**	**p = 0.0001**	**p = 0.001**	**p = 0.002**	**p = 0.003**	**p = 0.003**
Childhood trauma	0.003	0.005	0.005	**0.007**	**0.007**	**0.010**	0.002	0.004	0.005	**0.009**
	(-0.003, 0.008)	(-0.001, 0.010)	(-0.0004, 0.011)	**(0.002, 0.013)**	**(0.001, 0.014)**	**(0.003, 0.016)**	(-0.006, 0.009)	(-0.004, 0.011)	(-0.003, 0.014)	**(0.0001, 0.018)**
	p = 0.300	p = 0.083	p = 0.071	**p = 0.014**	**p = 0.033**	**p = 0.008**	p = 0.686	p = 0.373	p = 0.234	**p = 0.048**
Income	**-0.049**	**-0.055**	**-0.044**	**-0.050**	**-0.053**	**-0.056**	**-0.064**	**-0.083**	**-0.069**	**-0.088**
	**(-0.082, -0.016)**	**(-0.090, -0.021)**	**(-0.079, -0.009)**	**(-0.086, -0.014)**	**(-0.095, -0.010)**	**(-0.100, -0.013)**	**(-0.112, -0.016)**	**(-0.132, -0.034)**	**(-0.119, -0.019)**	**(-0.140, -0.037)**
	**p = 0.004**	**p = 0.002**	**p = 0.014**	**p = 0.007**	**p = 0.016**	**p = 0.012**	**p = 0.010**	**p = 0.001**	**p = 0.008**	**p = 0.001**
Constant	**-0.515**	**-0.494**	**-0.568**	**-0.542**	**-0.606**	**-0.601**	-0.414	-0.411	-0.372	-0.484
	**(-0.861, -0.170)**	**(-0.854, -0.134)**	**(-0.934, -0.203)**	**(-0.921, -0.162)**	**(-1.046, -0.165)**	**(-1.054, -0.149)**	(-0.910, 0.081)	(-0.918, 0.095)	(-0.899, 0.156)	(-1.026, 0.059)
	**p = 0.004**	**p = 0.008**	**p = 0.003**	**p = 0.006**	**p = 0.008**	**p = 0.010**	p = 0.103	p = 0.113	p = 0.168	p = 0.082
Observations (n)	**683**	**686**	**683**	**686**	**614**	**618**	**494**	**499**	**458**	**460**
R^2	**0.156**	**0.093**	**0.156**	**0.096**	**0.108**	**0.070**	**0.121**	**0.064**	**0.128**	**0.067**
Adjusted R^2	**0.150**	**0.087**	**0.150**	**0.090**	**0.101**	**0.062**	**0.112**	**0.055**	**0.118**	**0.057**

Supplementary Table 9 Cont.

Residual Std. Error	**0.759 (df = 677)**	**0.791 (df = 680)**	**0.802 (df = 677)**	**0.835 (df = 680)**	**0.924 (df = 608)**	**0.950 (df = 612)**	**0.906 (df = 488)**	**0.936 (df = 493)**	**0.907 (df = 452)**	**0.937 (df = 454)**
F Statistic	**25.004 (df = 5; 677)**	**13.982 (df = 5; 680)**	**25.011 (df = 5; 677)**	**14.515 (df = 5; 680)**	**14.743 (df = 5; 608)**	**9.159 (df = 5; 612)**	**13.385 (df = 5; 488)**	**6.756 (df = 5; 493)**	**13.279 (df = 5; 452)**	**6.570 (df = 5; 454)**
F Statistic (p-value)	**<0.001**	**<0.001**	**<0.001**	**<0.001**	**<0.001**	**<0.001**	**<0.001**	**<0.001**	**<0.001**	**<0.001**

Note: Results of linear regression models, not adjusted for multiple comparisons. Estimates are standardized betas; 95% Confidence Interval reported in brackets. Values in bold are statistically significant at a level p < 0.05 (two-sided). Results for PASS-process are shown for descriptive purposes only.

Supplementary Table 10. LORA: Prediction of stressor reactivity (SR) by perceived social support and perceived good stress recovery in the most stressor-exposed participants (top two terciles of mean E between B0 and B2), controlling for baseline (B0) covariates.

Predictor time point (battery)	B0						B1			
Outcome interval (SR score)	B0-B2 (~3.7 yrs)		B0-B1 (~1.9 yrs)		3 monitorings post B0 (~9 m)		B1-B2 (~1.6 yrs)		3 monitorings post B1 (~9 m)	
	Social Support	Stress recovery	Social Support	Stress recovery	Social Support	Stress recovery	Social Support	Stress recovery	Social Support	Stress recovery
Resilience Factor	**-0.118**<br>**(-0.183, -0.052)**<br>**p = 0.0005**	**-0.211**<br>**(-0.270, -0.151)**<br>**p < 0.00001**	**-0.113**<br>**(-0.182, -0.044)**<br>**p = 0.002**	**-0.227**<br>**(-0.290, -0.165)**<br>**p < 0.00001**	**-0.133**<br>**(-0.216, -0.050)**<br>**p = 0.002**	**-0.247**<br>**(-0.322, -0.172)**<br>**p < 0.00001**	**-0.266**<br>**(-0.347, -0.185)**<br>**p < 0.00001**	**-0.190**<br>**(-0.279, -0.101)**<br>**p = 0.00004**	**-0.248**<br>**(-0.332, -0.164)**<br>**p < 0.00001**	**-0.118**<br>**(-0.215, -0.021)**<br>**p = 0.018**
Age	-0.002<br>(-0.010, 0.007)<br>p = 0.698	0.0005<br>(-0.007, 0.008)<br>p = 0.908	-0.004<br>(-0.013, 0.004)<br>p = 0.342	-0.002<br>(-0.010, 0.006)<br>p = 0.604	-0.002<br>(-0.012, 0.009)<br>p = 0.755	0.001<br>(-0.009, 0.011)<br>p = 0.833	0.004<br>(-0.006, 0.015)<br>p = 0.418	0.004<br>(-0.006, 0.015)<br>p = 0.426	0.001<br>(-0.010, 0.012)<br>p = 0.902	0.003<br>(-0.009, 0.014)<br>p = 0.658
Sex	**0.392**<br>**(0.264, 0.520)**<br>**p = 0.000**	**0.301**<br>**(0.177, 0.426)**<br>**p = 0.00001**	**0.403**<br>**(0.267, 0.538)**<br>**p = 0.000**	**0.308**<br>**(0.177, 0.440)**<br>**p = 0.00001**	**0.382**<br>**(0.220, 0.544)**<br>**p = 0.00001**	**0.277**<br>**(0.120, 0.435)**<br>**p = 0.001**	**0.226**<br>**(0.053, 0.399)**<br>**p = 0.011**	**0.368**<br>**(0.190, 0.546)**<br>**p = 0.0001**	**0.204**<br>**(0.023, 0.386)**<br>**p = 0.028**	**0.332**<br>**(0.145, 0.519)**<br>**p = 0.001**
Childhood trauma	0.001<br>(-0.005, 0.007)<br>p = 0.775	0.002<br>(-0.003, 0.008)<br>p = 0.439	0.003<br>(-0.003, 0.010)<br>p = 0.281	0.004<br>(-0.001, 0.010)<br>p = 0.134	0.005<br>(-0.002, 0.013)<br>p = 0.149	**0.007**<br>**(0.0002, 0.014)**<br>**p = 0.046**	0.001<br>(-0.006, 0.008)<br>p = 0.797	-0.003<br>(-0.011, 0.006)<br>p = 0.540	0.005<br>(-0.003, 0.014)<br>p = 0.211	0.005<br>(-0.005, 0.014)<br>p = 0.337
Income	**-0.057**<br>**(-0.091, -0.023)**<br>**p = 0.002**	**-0.044**<br>**(-0.078, -0.010)**<br>**p = 0.011**	**-0.053**<br>**(-0.089, -0.017)**<br>**p = 0.005**	**-0.039**<br>**(-0.074, -0.003)**<br>**p = 0.034**	**-0.056**<br>**(-0.100, -0.013)**<br>**p = 0.012**	-0.042<br>(-0.085, 0.0003)<br>p = 0.052	**-0.063**<br>**(-0.110, -0.016)**<br>**p = 0.010**	**-0.076**<br>**(-0.125, -0.028)**<br>**p = 0.003**	**-0.070**<br>**(-0.120, -0.020)**<br>**p = 0.007**	**-0.086**<br>**(-0.137, -0.035)**<br>**p = 0.002**
Constant	**-0.392**<br>**(-0.759, -0.026)**<br>**p = 0.037**	**-0.404**<br>**(-0.758, -0.050)**<br>**p = 0.026**	**-0.449**<br>**(-0.837, -0.060)**<br>**p = 0.024**	**-0.446**<br>**(-0.820, -0.073)**<br>**p = 0.020**	**-0.516**<br>**(-0.973, -0.059)**<br>**p = 0.028**	**-0.535**<br>**(-0.977, -0.092)**<br>**p = 0.019**	-0.275<br>(-0.774, 0.224)<br>p = 0.281	-0.330<br>(-0.836, 0.175)<br>p = 0.201	-0.247<br>(-0.783, 0.288)<br>p = 0.366	-0.414<br>(-0.964, 0.137)<br>p = 0.142
Observations (n)	**688**	**689**	**688**	**689**	**618**	**619**	**495**	**497**	**458**	**458**
R^2	**0.084**	**0.128**	**0.082**	**0.131**	**0.067**	**0.111**	**0.125**	**0.081**	**0.126**	**0.073**
Adjusted R^2	**0.077**	**0.122**	**0.075**	**0.125**	**0.060**	**0.104**	**0.116**	**0.072**	**0.116**	**0.063**
Residual Std. Error	**0.793 (df = 682)**	**0.776 (df = 683)**	**0.840 (df = 682)**	**0.818 (df = 683)**	**0.949 (df = 612)**	**0.928 (df = 613)**	**0.901 (df = 489)**	**0.924 (df = 491)**	**0.906 (df = 452)**	**0.935 (df = 452)**
F Statistic	**12.531 (df = 5; 682)**	**20.040 (df = 5; 683)**	**12.191 (df = 5; 682)**	**20.679 (df = 5; 683)**	**8.842 (df = 5; 612)**	**15.339 (df = 5; 613)**	**13.990^{***} (df = 5; 489)**	**8.673 (df = 5; 491)**	**13.039 (df = 5; 452)**	**7.163 (df = 5; 452)**
F Statistic (p-value)	**<0.001**	**<0.001**	**<0.001**	**<0.001**	**<0.001**	**<0.001**	**<0.001**	**<0.001**	**<0.001**	**<0.001**

Note: Results of linear regression models, not adjusted for multiple comparisons. Estimates are standardized betas; 95% Confidence Interval reported in brackets. Values in bold are statistically significant at a level p < 0.05 (two-sided.

Supplementary Table 17. LORA: Results of covariate selection.

Variables	p value	p<0.2
age in years	0.249	Yes*
sex	<0.001	Yes*
relationship status	0.376	No
employment status	.801	No
education	.966	No
persons household income[1]	.284	No
household income[2]	<0.001	Yes
life events score[3]	.283	No
childhood trauma[4]	<0.001	Yes
alcohol use[5]	.319	No

Note: [1] number of persons contributing to your household income; [2] average monthly net income of your household; [3] Summary of life events score before baseline; [4] Childhood trauma questionnaire (CTQ); [5] Alcohol use disorder identification test (AUDIT)
*were included independent of p value.

Interventional sample: RESPOND-RCT Spain

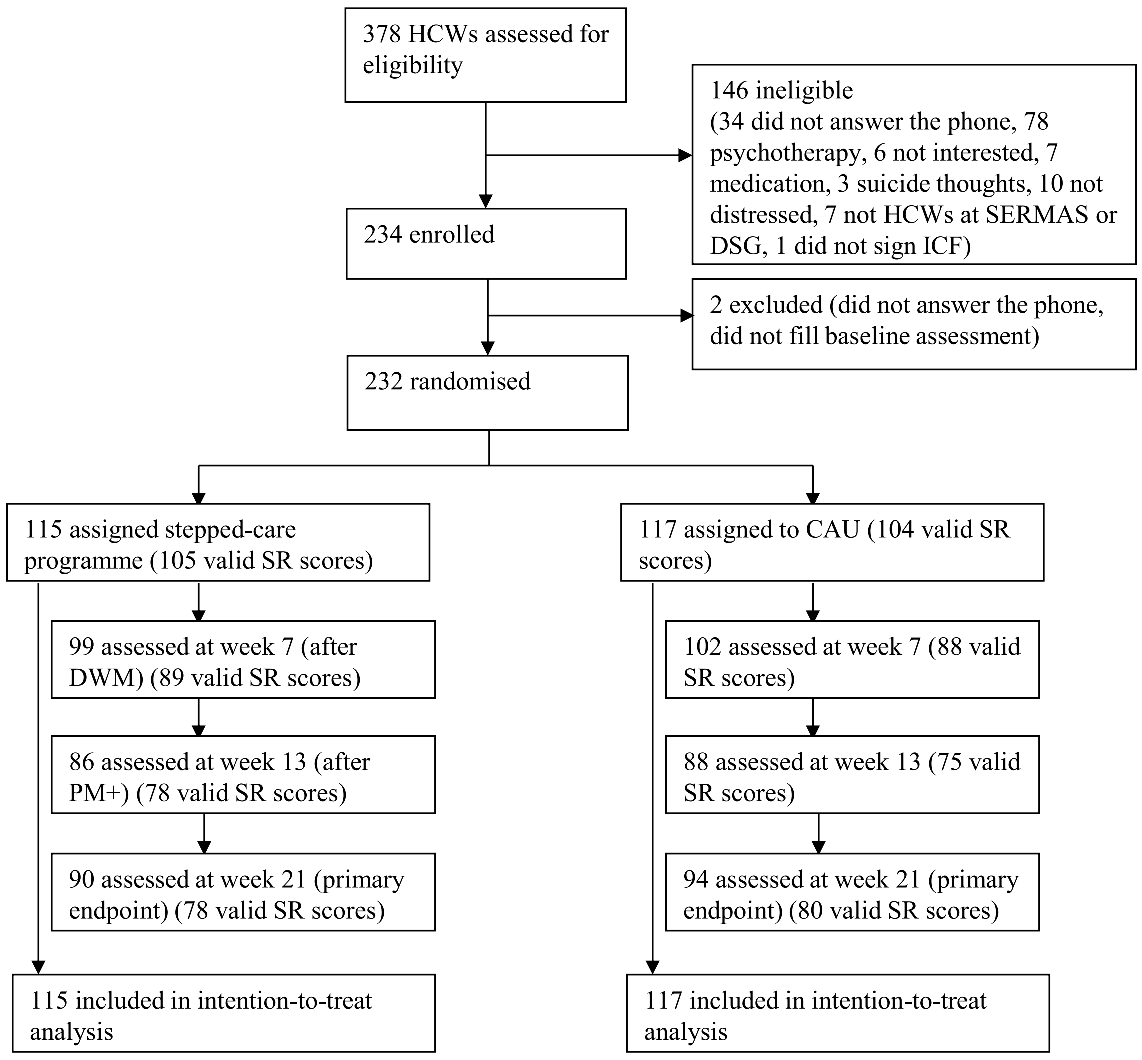

Supplementary Figure 1. CONSORT Flow Diagram of RESPOND-RCT Spain.

Note: HCWs = healthcare workers; SERMAS = Servicio Madrileño de Salud [Madrid Health Department]; DSG = Department de Salut de la Generalitat [Catalonia Health Department]; SR = stressor reactivity; CAU = care as usual

Supplementary Table 11. RESPOND-RCT Spain: Life events (LE) list.

We would like to ask you about life events that you experienced. Please consider which of these events happened in the past 2 months or since the last time we asked you. Please rate their impact on you. [(0) This situation did not happen; (1) No impact at all; (2) A little impact; (3) Moderate impact; (4) Severe impact]

Item	Question
LE_1	Serious illness, accident or diagnosis of disease experienced by me or a close person (e.g. family member or close friend)
LE_2	Death of a close person (e.g. family member or close friend)
LE_3	Break up/separation/divorce from partner

Supplementary Table 12. RESPOND-RCT Spain: Daily hassles (DH) lists.

We would like to ask you about annoyances and hassles as may occur in daily life.
The list below contains possible situations. Please only consider the last 14 days including today.
Please read each item on the list and consider if it happened to you: ([0-3] 'did not happen/almost never' 'sometimes' 'often' to '(nearly) every day')

	Item	Question
General stressors		
1	EG_1	Conflict or disagreement at work (for example: with colleagues or boss)
2	EG_2	Conflict or disagreement with close persons (for example: family member or close friend)
3	EG_3	Lack of help/support from others
4	EG_4	Financial problems (not having enough money for basic services, emergencies or special wishes)
5	EG_5	High demands/high workload/Time pressure
6	EG_6	Bad personal news (for example: rejection letter, being fired, friend moving out of town, etc.)
Pandemic-related stressors		
7	EC_1	Being at increased risk of a COVID-19 infection
8	EC_2	(Feeling) restricted to leave your home or having to quarantine.
9	EC_3	Not being able to perform leisure activities
10	EC_4	Lack of social contact
11	EC_5	Less physical activity then usual
Population-specific stressors		
12	EH_1	Patients under your care have died due to COVID-19
13	EH_2	People you know have been hospitalized due to COVID-19
14	EH_3	Change in duties or unclear duties and protocols
15	EH_4	Difficulty combining social life with work

Supplementary Table 13. RESPOND-RCT Spain: demographics and group comparison.

		Group	
	Overall, N = 232	Control, n = 117	Intervention, n = 115
Age, M (SD)	37.5 (10.3)	37.1 (10.4)	37.9 (10.1)
Gender, n (%)			
Female	200 (86%)	99 (85%)	101 (88%)
Male	32 (14%)	18 (15%)	14 (12%)
Educational level			
Secondary	1 (0.4%)	0 (0%)	1 (0.9%)
Technical-professional	41 (18%)	18 (15%)	23 (20%)
University	190 (82%)	99 (85%)	91 (79%)
Type of job			
Physician	50 (22%)	28 (24%)	22 (19%)
Nurse	130 (56%)	66 (57%)	64 (56%)
Nursing technician	29 (13%)	12 (10%)	17 (15%)
Administration	6 (2.6%)	1 (0.9%)	5 (4.3%)
Other	16 (6.9%)	9 (7.8%)	7 (6.1%)
Job facility			
Hospital facilities	147 (63%)	72 (62%)	75 (65%)
Primary care facilities	68 (29%)	35 (30%)	33 (29%)
Specialised care facilities	5 (2.2%)	3 (2.6%)	2 (1.7%)
Emergencies	10 (4.3%)	6 (5.1%)	4 (3.5%)
Other	2 (0.9%)	1 (0.9%)	1 (0.9%)
Frontline worker (ever)	215 (93%)	108 (92%)	107 (93%)
COVID-19 infection (ever)	137 (59%)	69 (59%)	68 (60%)
Site			
Madrid	110 (47%)	55 (47%)	55 (48%)
Catalonia	122 (53%)	62 (53%)	60 (52%)
Anxiety/Depression symptoms (PHQ-ADS score, 0–48), M (SD)	20.5 (8.5)	20.2 (8.8)	20.8 (8.1)
Depression symptoms (PHQ-9 score, 0–27)	10.3 (4.8)	10.0 (4.9)	10.6 (4.6)
Anxiety symptoms (GAD-7 score, 0–21)	10.2 (4.4)	10.2 (4.5)	10.2 (4.2)

Posttraumatic stress symptoms (PCL-5 score, 0–32)	12.9 (6.2)	12.7 (6.2)	13.1 (6.3)
Probable major depressive disorder (PHQ-9 > 9), n (%)	124 (53%)	56 (48%)	68 (59%)
Probable anxiety disorder (GAD-7 > 9)	134 (58%)	70 (60%)	64 (56%)

Note. M = mean, SD = standard deviation, PHQ-ADS = Patient Health Questionnaire – Anxiety and Depression Scale, PHQ-9 = Patient Health Questionnaire, GAD-7 = Generalised Anxiety Disorder Questionnaire, PCL-5 = PTSD checklist for the DSM-5

Supplementary Table 14. RESPOND-RCT Spain: Stressor exposure per assessment time point and category (means and standard deviations).

	T0 Mean (sd)	T1 Mean (sd)	T2 Mean (sd)	T3 Mean (sd)
Life events	2.17 (0.85)	0.32 (0.62)	0.30 (0.58)	0.30 (0.62)
General stressors	5.91 (2.65)	5.05 (2.79)	4.37 (2.85)	4.25 (2.65)
Pandemic-related stressors	5.72 (3.09)	4.09 (2.82)	3.74 (2.59)	3.76 (2.53)
Population-specific stressors	4.10 (2.29)	3.06 (2.10)	2.54 (1.97)	2.46 (1.99)

Note: sd = standard deviation.

Supplementary Table 15a. RESPOND-RCT Spain: Effect of the intervention on SR.

Results are reported both for a baseline-adjusted linear mixed model (including the baseline value of SR as a fixed effect) as well as fully adjusted linear model (controlling for age, gender, level of education, additionally to SR at baseline), as were done for the pre-registered primary anayles. We report estimated marginal means (EMM) comparison between intervention and control group and standardized effect sizes (SES) of the intervention on SR and their respective 95% confidence intervals.

		Baseline-adjusted models			Fully adjusted models		
	Time Point	Control (EMM)	Intervention (EMM)	Cohen's d	Control (EMM)	Intervention (EMM)	Cohen's d
SR	1	0.21 (0.02,0.39)	-0.10 (-0.28,0.09)	0.43 (0.10,0.77)	0.27 (0.05,0.49)	-0.05 (-0.30,0.19)	0.42 (0.08,0.75)
	2	0.09 (-0.14,0.32)	-0.40 (-0.63,-0.17)	0.67 (0.25,1.09)	0.15 (-0.10,0.40)	-0.36 (-0.63,-0.08)	0.66 (0.24,1.08)
	3	0.06 (-0.17,0.29)	-0.30 (-0.52,-0.07)	0.50 (0.07,0.92)	0.11 (-0.14,0.36)	-0.26 (-0.55,0.04)	0.48 (0.06,0.90)
Note: EMM= report estimated marginal means							

Supplementary Table 15b. RESPOND-RCT Spain: Effect sizes for the effect of the intervention on E, P and PAS, as well as the effect of PAS at baseline on SR.

We report estimated marginal means (EMM) comparison between intervention and control group and standardized effect sizes (SES) of the intervention on SR and their respective 95% confidence intervals.

Effect	Time Point	Control (EMM)	Intervention (EMM)	Cohen's d
Intervention on E	3	12.16 (10.54, 13.79)	9.38 (7.8, 10.96)	0.65 (0.23, 1.07)
Intervention on E	2	12.57 (10.85, 14.28)	9.43 (7.71, 11.14)	0.74 (0.29, 1.18)
Intervention on E	1	13.61 (12.03, 15.19)	11.31 (9.61, 13)	0.54 (0.11, 0.97)
Intervention on P	3	18.2 (16.02, 20.38)	13.83 (12, 15.67)	0.78 (0.35, 1.22)
Intervention on P	2	18.81 (16.58, 21.05)	13.17 (11.33, 15)	1.01 (0.57, 1.46)
Intervention on P	1	20.3 (18.49, 22.12)	16.96 (15.19, 18.73)	0.6 (0.23, 0.97)
Intervention on PAS	3	29.99 (28.38, 31.61)	32.26 (30.77, 33.75)	-0.62 (-1.15,-0.08)
Intervention on PAS	2	29.25 (27.75, 30.76)	32.66 (31.24, 34.08)	-0.93 (-1.43,-0.43)
Intervention on PAS	1	29.28 (27.76, 30.8)	31.04 (29.7, 32.37)	-0.48 (-0.96, 0)
PAS at baseline on SR	3	0.24 (-0.07, 0.55)	-0.16 (-0.47, 0.15)	0.55 (0.1, 1)
PAS at baseline on SR	2	0.21 (-0.09, 0.52)	-0.26 (-0.54, 0.03)	0.65 (0.21, 1.09)

PAS at baseline on SR	1	0.33 (0.05, 0.61)	0.06 (-0.21, 0.33)	0.38 (0.03, 0.73)
Note: EMM= report estimated marginal means				

Supplementary Table 18. RESPOND-RCT Spain: Results of covariate selection.

Variables	p value	p<0.2
Age	0.545	Yes*
Gender	0.850	Yes*
Education	0.160	Yes
Prior use of mental health services	0.490	No

*were included independent of p value.

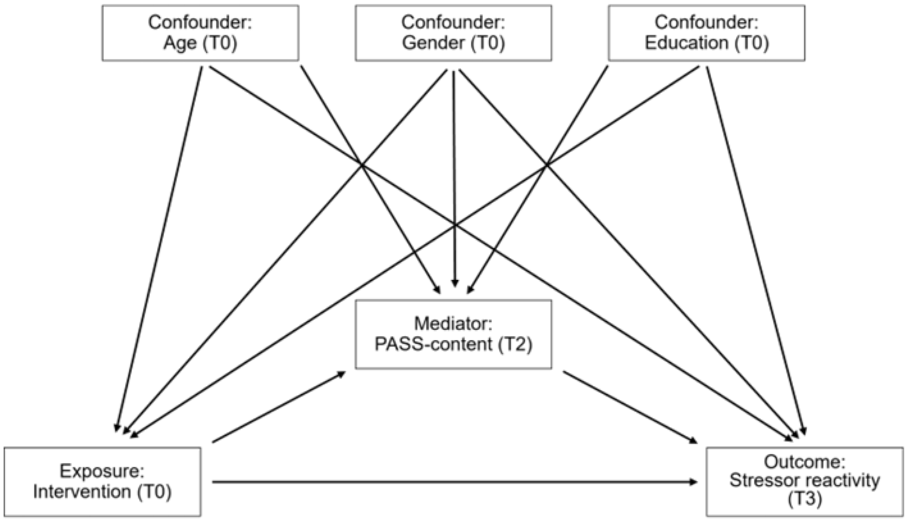

Supplementary Figure 2. RESPOND-RCT Spain: Directed acyclic graph showing the hypothetical causal pathways.

Supplementary Table 19. RESPOND-RCT Spain: Comparison of mediation models.

		Method	Baseline measures	Imputation	Estimate	Std.error	95% CL	95% CIU	P.val
Cde	1	Reg	Yes	Yes	-3.06	2.79	-8.31	2.37	0.28
	2	Reg	Yes	No	-5.07	2.94	-10.62	0.91	0.10 .
	3	Weight	Yes	No	-5.07	3.09	-10.96	0.91	0.11
pnde	1	Reg	Yes	Yes	-3.06	2.79	-8.31	2.37	0.28
	2	Reg	Yes	No	-5.07	2.94	-10.62	0.91	0.10 .
	3	Weight	Yes	No	-5.58	2.99	-11.29	0.33	0.07 .
Tnde	1	Reg	Yes	Yes	-3.06	2.79	-8.31	2.37	0.28
	2	Reg	Yes	No	-5.07	2.94	-10.62	0.91	0.10 .
	3	Weight	Yes	No	-4.75	3.26	-10.76	1.40	0.15
Pnie	1	Reg	Yes	Yes	-2.31	1.30	-5.24	-0.09	0.04 *
	2	Reg	Yes	No	-2.43	1.44	-5.54	-0.10	0.04 *
	3	Weight	Yes	No	-2.80	1.47	-6.01	-0.19	0.04 *
Tnie	1	Reg	Yes	Yes	-2.31	1.30	-5.24	-0.09	0.04 *
	2	Reg	Yes	No	-2.43	1.44	-5.54	-0.10	0.04 *
	3	Weight	Yes	No	-1.97	1.45	-5.06	0.81	0.18
Te	1	Reg	Yes	Yes	-5.37	2.54	-10.18	-0.28	0.04 *
	2	Reg	Yes	No	-7.49	2.80	-12.86	-2.08	0.01 **
	3	Weight	Yes	No	-7.55	2.94	-13.21	-1.59	0.01 *
Pm	1	Reg	Yes	Yes	0.47	16.39	-1.35	2.23	0.16
	2	Reg	Yes	No	0.32	0.62	0.003	1.23	0.05 *
	3	Weight	Yes	No	0.26	0.53	-0.19	1.01	0.18

Note: Cde= Controlled direct effect. Pnde=pure natural direct effect. Tnde= Total natural direct effect. Pnie = Purue natural indirect effect. Tnie= Total natural indirect effect. Te= Total effect. Pm= Proportion mediation. Reg= Regression-based approach. Weight= Weight-based approach. CIL = Lower confidence interval. CIU= Upper confidence interval. Std error = standard error. P val = p value.

References

1. Kalisch, R., Müller, M. B. & Tüscher, O. A conceptual framework for the neurobiological study of resilience. *Behav Brain Sci* **38**, e92 (2015).
